# Risk Factors for Severe Respiratory Syncytial Virus Infection in Hospitalized Children

**DOI:** 10.3390/v15081713

**Published:** 2023-08-09

**Authors:** Małgorzata Kobiałka, Teresa Jackowska, August Wrotek

**Affiliations:** 1 Department of Pediatrics, Bielanski Hospital, 01-809 Warsaw, Poland; 2 Department of Pediatrics, Centre of Postgraduate Medical Education, 01-813 Warsaw, Poland

**Keywords:** respiratory syncytial virus, bronchiolitis, clinical course, risk factors, pediatrics, children

## Abstract

Background: RSV often leads to hospitalization, and accurate knowledge of risk factors is crucial. Methods: We retrospectively analyzed laboratory-confirmed RSV hospitalizations regarding pregnancy factors, birth status, cigarette smoke exposure, nutrition, social conditions, clinical presentation, and severe disease defined as a need for passive oxygen therapy (pO2Tx), the presence of pneumonia, respiratory failure, intensive care unit (ICU) transfer, and prolonged hospitalization. Results: A univariate analysis included 594 children (median age 4 months) and revealed a pO2Tx relationship with age ≤ 3 months (OR = 1.56), prematurity (OR = 1.71), being born during RSV season (OR = 1.72), smoke exposure during pregnancy (both parents (OR = 2.41, father (OR = 1.8)), dyspnea (OR = 5.09), and presence of apnea (OR = 5.81). Pneumonia was associated with maternal smoke exposure (OR = 5.01), fever (OR = 3.92), dyspnea (OR = 1.62), history of aspiration (OR = 4.63), and inversely with age ≤ 3 months (OR = 0.45). Respiratory failure was associated with prematurity (OR = 3.13) and apnea (OR = 18.78), while the lower odds were associated with older age (OR = 0.57 per month) and presence of fever (OR = 0.11). ICU transfer was associated with apnea (OR = 17.18), but an inverse association was observed with age (OR = 0.54) and fever (OR = 0.11). A prolonged hospital stay was associated with prematurity (OR = 1.76), low birth weight (OR = 2.89), aspiration (OR = 4.93), and presence of fever (OR = 1.51). Conclusions: Age (up to 3 months), prematurity, and presence of apnea are risk factors for a severe RSV course.

## 1. Introduction

Respiratory syncytial virus (RSV) is one of the most common etiological factors of acute lower respiratory tract infections (ALRI) in children, typically presenting as acute bronchiolitis, pneumonia, or bronchitis [1]. Worldwide, RSV is responsible for approximately 33 million cases per year in children under 5 years of age, leading to hospitalization in approximately 11% of cases [2]. The burden of RSV disease in infants is high, with global estimates reporting a median of 45% of cases under 5 years of age affecting infants, while in Europe it is estimated to be 2–2.5 times higher than in the US, and French data show that RSV accounts for approximately 28% of all-cause hospitalizations, generating a total cost of 124.1 million euros per year in France, with a strong upward trend [3,4,5]. In Poland, the mean annual estimated RSV hospitalization rate reached 267.5 per 100,000, and the majority of cases was observed in children < 1 year of age (1132.1 per 100,000), with those under 6 months of age accounting for over 60% of the total number of cases [6]. In Poland, as in many other European countries, a general trend of increase in RSV incidence and hospitalization has been reported, although this may be related to improvements in the diagnostic approach. Some authors even claim that in fact the rate of hospitalization remains stable, but the true magnitude of RSV epidemiology is being unravelled [6,7,8]. Interestingly, due to the introduction of public health measures related to the SARS-CoV-2 pandemic, including social distancing or wearing masks, a temporary decrease in the number of RSV cases was observed, but this was followed by a sharp increase in both RSV incidence and hospitalizations [9,10,11,12]. Although the mortality rate is rather low in high-income countries (e.g., 0.08% of all RSV patients in Poland), RSV is responsible for more than 100,000 deaths in children aged 0–60 months worldwide, mostly in low- and middle-income countries [2,6].

The socioeconomic burden of RSV is enormous and extends far beyond the youngest group of patients, as RSV is also one of the most important etiological agents of ALRI in adults, including the elderly, with a high in-hospital case fatality rate, even in high-income countries [13,14,15]. All these causes lead to an urgent need for effective prophylactic measures, and a number of ongoing studies are being conducted. Currently, the most commonly tested protective strategies include maternal immunization, infant immunization, or the use of long-lasting monoclonal antibodies [16,17,18]. The use of palivizumab, a recombinant humanized monoclonal antibody targeting the fusion (F) protein of RSV, is limited to selected risk groups, mainly preterm infants less than 35 weeks of gestational age, up to 6 months of age, and certain comorbidities, and because of its relatively high cost, it is subject to local regulations that determine the extent of its prophylactic use [18,19,20].

The most vulnerable groups deserve special attention because of the risk associated with RSV disease, and a number of studies and meta-analyses have been conducted and published on risk factors. These include prematurity, low birth weight, young age (less than 6 months and especially less than 3 months), congenital heart disease, and comorbidities, such as respiratory and/or cardiovascular disease, neurological disease, and blood or liver disease [21,22,23]. A recent meta-analysis by Shi and colleagues emphasized the need for more studies using multivariable analysis to elucidate risk factors associated with poor outcome [22]. Furthermore, it must be emphasized that under current circumstances, only about 2% of patients qualify for prophylactic use of palivizumab, while a large number of severe RSV cases are seen in otherwise healthy populations with no predictive factors for severe disease course or hospitalization [18,24]. Similarly, in Poland, a broad prophylactic program with the use of palivizumab has been successfully introduced, thus decreasing the number of hospitalizations of the high-risk patients [25]. The rationale behind the study was to verify which of the aforementioned risk factors can be confirmed in everyday clinical practice from a perspective of a mid-size pediatric ward, taking into account that the frequency of RSV disease might be lowered in particular risk groups thanks to the prophylactic programs. The choice of the risk groups was based upon the most recent literature, additionally an analysis on clinical presentation was performed [21,22,23].

In this study, we analyzed the presence of severe disease, i.e., the need for passive oxygen therapy (pO2Tx), the presence of pneumonia, respiratory failure, intensive care unit (ICU) transfer, prolonged (i.e., above median) length of stay, or an unfavorable outcome. We aimed to evaluate multiple variables as possible risk factors in a general pediatric population hospitalized for a RSV episode in the general pediatric ward, focusing not only on underlying conditions, prematurity, or age but also on clinical presentation (signs/symptoms), laboratory studies (serum inflammatory markers and capillary blood gas), as well as social conditions and feeding methods, using both univariate and multivariate models.

## 2. Materials and Methods

### 2.1. Study Protocol and Data Collection

The study was a retrospective analysis of a cohort of patients hospitalized for laboratory-confirmed lower respiratory tract RSV infection at the Pediatric Department of Bielanski Hospital in Warsaw; the Bielanski Hospital is a multidisciplinary mid-size hospital serving over 250,000 citizens, with a pediatric population of approximately 50,000; the Pediatric Department has 35 beds and around 2500 children (aged 0–18 years old) are hospitalized each year. The medical records of patients hospitalized in the period between January 2010 and January 2019 were searched for one of the following ICD-10 (10th Revision of the International Classification of Diseases) final diagnoses: J12.1-RSV pneumonia, J20.5-RSV bronchitis, and J21-RSV bronchiolitis. Initially, enrolled charts were reviewed for inclusion and exclusion criteria.

### 2.2. Inclusion Criteria

Inclusion criteria were age (patients aged 0 to 2 years), hospitalization in the pediatric unit, final diagnosis of lower respiratory tract infection (pneumonia, bronchitis, bronchiolitis), and laboratory confirmation of RSV infection. Only patients with community-acquired infections were included. Similarly, a clinical diagnosis of RSV infection had to be accompanied by laboratory confirmation, i.e., a positive result of a rapid antigen diagnostic test (Alere BinaxNOW, Alere Scarborough Inc.; Scarborough, ME, USA; and NADAL RSV Test; nal von minden GmbH, Moers, Germany) or RT-PCR (RSV Xpert Xpress Flu/RSV GeneXpert, Cepheid, Sunnyvale, CA, USA). Diagnostic tests were performed on nasopharyngeal swab samples according to the manufacturer’s instructions. In accordance with the official Polish guidelines for the management of respiratory tract infections, bronchitis was defined on the clinical basis of the presence of cough (productive or unproductive) and wheezing or rales on examination, while bronchiolitis was defined as the first episode of bronchial obstruction accompanied by wheezing/rales on physical examination, expiratory dyspnea, and/or hypoxia, while pneumonia was defined as at least 2 of the pneumonia signs or symptoms, i.e., fever of at least 38 degrees Celsius, cough, tachypnea (according to age groups, i.e., >60 breaths/minute in 0 < 2 months, >50 in 2–12 months, >40 breaths/minute in >12 months), and retraction of intercostal spaces together with crackles or bronchial murmur or dull percussion sound [25]. All diagnoses were made by the physicians responsible for the patient’s care and were verified retrospectively using the patient’s medical record. In addition, the diagnosis of pneumonia was confirmed by the result of an imaging study (chest X-ray or lung ultrasound) consistent with pneumonia, i.e., the presence of consolidations, densities (linear or patchy), parenchymal infiltrates, and/or pleural effusion in the case of a chest X-ray, and the presence of consolidations, hypoechogenic lesions, local absence or hypoechogenic pleural line, hyperechogenic areas within consolidations, or impaired lung sliding in the case of lung ultrasound.

### 2.3. Exclusion Criteria

We excluded patients with diagnosed/suspected immune deficiency syndrome, regardless of its origin (hereditary or acquired). If a patient presented signs/symptoms of respiratory infection after 48 h of hospitalization, we excluded the patient because of suspicion of nosocomial infection. In the case of a lack of complete knowledge about the clinical course of the disease, i.e., the patient was discharged on request, the patient was excluded from further analysis. If there was only partial but not crucial missing information, e.g., missing medical history for minor questions, the patients were included, but the data were treated as missing.

### 2.4. Clinical Data

We collected sociodemographic data (sex, age, younger age defined as ≤3 months) as well as data on risk factors related to pregnancy/perinatal period (duration of pregnancy, prematurity defined as birth before 37 weeks of gestation, birth weight, including low birth weight defined as weight below 2500 g, Apgar score, being born during the RSV peak season, i.e., from October to April, cigarette smoke exposure during pregnancy (including smoking by the mother or father only and smoking by both parents)), maternal age, current feeding method (breastfeeding versus mixed versus modified milk), presence of congenital heart disease, neurological disorder, trisomy 21, poorer social conditions (defined as a higher number of persons per room than the median for the whole study group), number of siblings, and clinical presentation. The latter was based on a parent/caregiver history and included the presence of fever (i.e., body temperature of at least 38 degrees Celsius), duration of fever, highest recorded fever, duration of symptoms before hospitalization, cough, dyspnea, apnea, history of aspiration, oxygen saturation in blood by pulse oximetry (%Sat), and laboratory results—serum inflammatory markers and capillary blood gas analysis (CBG). The inflammatory markers included C-reactive protein (CRP), procalcitonin (PCT), white blood cell count (WBC), and absolute neutrophil count (ANC), while the CBG parameters included acidosis (defined as pH < 7.35), pCO_2_ (and hypercapnia defined as pCO_2_ > 45 mmHg) and oxygen saturation (SatO_2_).

CRP levels were determined using the Cobas 6000 analyzer (Roche Diagnostics Ltd., Rotkreuz, Switzerland), while procalcitonin was measured using the Cobas e411 (until 4 August 2016) and Cobas 6000 (from 5 August 2016), both from Roche Diagnostics Ltd., Rotkreuz, Switzerland. The detection limits were 0.1 mg/L for CRP and 0.02 ng/mL for procalcitonin. WBC and ANC were measured by Sysmex XT2000i until 9 April 2014, and by Sysmex XN1000/Sysmex XN550 (Sysmex Corporation, Kobe, Japan) since 10 April 2014. CBG parameters were determined using the Roche Cobas b 121 and b 221 analyzer (Roche Diagnostics Ltd., Rotkreuz, Switzerland) until 26 January 2016, and using the Siemens RAPIDLab 348EX Blood Gas System (Siemens Healthcare Diagnostics, Marburg, Germany) since 27 January 2016; we included pH, partial pressure of carbon dioxide (pCO_2_), and oxygen saturation (SatO_2_) in the analysis.

### 2.5. End-Points

Our main objective was to assess the importance of risk factors for the presence of severe RSV disease. Severe disease was defined as the use of passive oxygen therapy (pO2Tx), the presence of pneumonia, respiratory failure, ICU transfer, prolonged (i.e., above median) length of stay, or an unfavorable outcome. According to the Polish guidelines on bronchiolitis treatment, passive oxygen therapy was initiated if a patient’s blood oxygen saturation decreased below 90% with the aim of reaching at least 90% of saturation [25]. In Poland, recommended oxygen delivery modes are nasal prongs and oxygen face masks, while at our ward, nasal prongs are the method of choice for low-flow oxygen therapy; in the analyzed period, high-flow nasal cannula (HFNC) had not been used.

### 2.6. Statistical Analysis

Categorical variables were presented as a number of observations and a proportion in corresponding groups, while numerical variables were presented as mean and standard deviation or median and interquartile range (first and third quartile), depending on the normality of the distribution. Normality of distribution was assessed using the Shapiro–Wilk test, as well as skewness and kurtosis. Homogeneity of variance was assessed using Levene’s test.

Group comparisons between categorical variables were performed using the Pearson chi-squared test or the Fisher exact test, as appropriate. Group comparisons for numerical variables were performed using *t*-Student test for independent groups, *t*-Welch test for independent groups, or U Mann–Whitney test, as appropriate.

A two-step logistic regression analysis was performed to identify significant predictors of selected diseases/conditions. First, univariate models were run for all parameters in the study to indicate which variables have a significant effect in an isolated setting. The *p*-values obtained from the univariate models were used to initially select potentially significant parameters for the second phase of regression analysis—multivariate models. Multivariate regression models were verified using Negelkerky R2 and Hosmer–Lemeshow good of fitness (GOF) test to assess the quality of fit. Variance inflation factor (VIF) parameters were used to identify potential multicollinearity. All statistical calculations were performed at a significance level of alpha = 0.05. Statistical analysis was performed using R software, version R-4.1.2.

The study was approved by the local Ethics Committee at the Center for Postgraduate Medical Education, Warsaw, Poland (approval number 32/PB/2019 issued on 13 March 2019). The study was conducted in accordance with the Declaration of Helsinki and its subsequent amendments; the procedures were in accordance with standard procedures, and no additional type of study/test/intervention was associated with the study. Due to the retrospective nature of the study, informed consent of the patient’s legal guardian was not required.

## 3. Results

**Figure 1:**
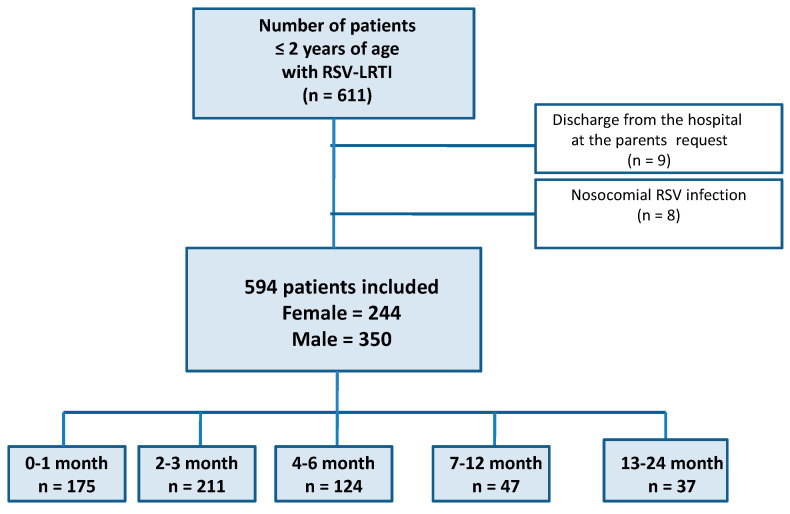
Flowchart of the patients in the study. RSV—respiratory syncytial virus; LRTI—lower respiratory tract infections.

A total of 611 children were diagnosed with RSV LRTI between January 2010 and January 2019; 9 children were discharged on request and 8 patients were excluded due to nosocomial infections; an increase in the number of diagnosed cases was observed (2010—17 cases, 2011—31, 2012—32, 2013—86, 2014—66, 2015—60, 2016—50, 2017—104, 2018—100, January 2019—48 cases). Finally, a group of 594 laboratory-confirmed RSV LRTI hospitalizations was included (a flowchart of patients in the study is shown in Figure 1). The study group included 244 females (41.1%) and 350 males (58.9%), ranging in age from 7 days to 24 months, with a mean age of 4.04 (±4.56) months; detailed baseline characteristics of the study group are shown in Table 1. A total of 16 patients were transferred to the ICU (15 for respiratory failure, one for recurrent apnea), and no deaths occurred, so the latter part of the analysis was not performed.

### 3.1. Risk Factors for a Passive Oxygen Therapy

The odds of using pO2Tx were higher in children aged ≤ 3 months (OR = 1.56) and preterm infants (OR = 1.71), those born during the infectious season (OR = 1.72), and those exposed to nicotine during pregnancy, to a greater extent when both parents smoked cigarettes (OR = 2.41) and when the father smoked cigarettes (OR = 1.8); it was irrelevant in the case of maternal exposure to nicotine. Patients who received pO2Tx were more likely to present with dyspnea on admission (OR = 5.09), to have apnea (OR = 5.81), and to have lower saturation on pulse oximetry (OR = 0.78). Also, a lower oxygen saturation on CBG was associated with higher odds of pO2Tx (OR = 0.87), as were pCO_2_ (OR = 1.1) and hypercapnia (OR = 2.66), but not acidosis.

The multivariate model showed that breastfeeding was a protective factor against the need for passive oxygen therapy (OR = 0.49), while patients with a higher Apgar score at birth had an increased likelihood of pO2Tx (OR = 1.6). Patients who presented with dyspnea (OR = 5.22) had higher odds of pO2Tx, while pulse oximetry saturation (OR = 0.83) and CBG saturation (OR = 0.87) were (inversely) related to the need for oxygen therapy. The multivariate regression model was verified with Nagelkerky R2, which reached 34.8%, and a Hosmer–Lemeshow GOF test with the result of *p* = 0.256, confirming that the model fit was satisfactory (Table 2 and Table 3).

### 3.2. Risk Factors for Pneumonia

Both univariate and multivariate models indicated a significant risk factor for pneumonia. In the univariate model, patients with pneumonia were older (OR = 1.1) and had a history of maternal smoke exposure (OR = 5.01). A clinical presentation differed between patients with and without pneumonia, and those with a history of aspiration (OR = 4.63), fever (OR = 3.92), longer duration of fever (OR = 1.54 per day), longer duration of symptoms (OR = 1.1), and dyspnea (OR = 1.62) had higher odds of being diagnosed with pneumonia. Among laboratory parameters, a significant association was found for CRP (OR = 1.07 per 1 mg/L increase), PCT (OR = 1.44 per 1 ng/mL), WBC (OR = 1.05 per 1000/uL), ANC (OR = 1.15 per 1000/uL), and hypercapnia (OR = 2.06).

The multivariate model showed statistical significance for history of aspiration (OR = 40.75), duration of fever (OR = 1.7 per day), CRP (OR = 1.06), PCT (OR = 0.61), and hypercapnia (OR = 4.36). The multivariate regression model was verified with Nagelkerky R2, which was 36.0%, and Hosmer–Lemeshow GOF test, which gave the results of *p* = 0.409, confirming that the model fit was satisfactory (Table 4 and Table 5).

### 3.3. Risk Factors for Respiratory Failure

In the univariate model, we observed that the higher odds of respiratory failure were associated with prematurity (OR = 3.13) and apnea (OR = 18.78), while the lower odds were associated with older age (OR = 0.57 per month) and the presence of fever (OR = 0.11). Worse CBG parameters on admission were also correlated with the risk of respiratory failure, including higher pCO_2_ (OR = 1.16) and the presence of hypercapnia (OR = 7.46), as well as a lower oxygen saturation (OR = 0.88).

The multivariate model showed that among all the variables mentioned above, only apnea was independently associated with higher odds of respiratory failure (OR = 29.4). A satisfactory multivariate regression model fit was confirmed by Nagelkerky R2 (37.9%) and Hosmer–Lemeshow GOF (*p* = 0.058) (Table 6 and Table 7).

### 3.4. Risk Factors for Intensive Care Unit Transfer

In the univariate model, one of the risk factors for the ICU transfer was apnea (OR = 17.18), and an inverse relationship was observed in the case of age (OR = 0.54), fever (OR = 0.11), and CBG parameters: pCO_2_ (OR = 1.16) and hypercapnia (OR = 6.7), and oxygen saturation (OR = 0.87).

In the multivariate model, male gender was associated with an increased risk of the ICU transfer (OR = 8.31), together with apnea (OR = 21.99), CRP (OR = 1.17), and worse CBG saturation (OR = 0.83). The multivariate regression model fit was satisfactory, with Nagelkerky R^2^ = 45.1% and Hosmer–Lemeshow GOF *p* = 0.96, (Table 8 and Table 9).

### 3.5. Prolonged Hospitalization

The univariate analysis of the risk factors of prolonged hospitalization (i.e., above the median, which reached 9 days in the study group) pointed to prematurity (OR = 1.76), low birth weight (OR = 2.89), aspiration (OR = 4.93), presence of fever (OR = 1.51), and CBG parameters: pCO_2_ (OR = 1.04 and OR = 2.24 in the case of hypercapnia) and oxygen saturation (OR = 0.96).

The multivariate model showed no statistical significance in terms of a prolonged hospital stay with Nagelkerky R2 = 13.3% and Hosmer–Lemeshow GOF *p* = 0.136, (Table 10 and Table 11).

## 4. Discussion

The aim of this study was to evaluate the importance of risk factors for severe RSV disease in daily clinical practice in a general pediatric ward. Our data added to the growing body of research, revealing both confirmation and lack of confirmation depending on the factors analyzed and the type of analysis.

The most important issue from a clinical point of view is the risk of respiratory failure and ICU admission; our results are similar for these two endpoints since the vast majority of patients (15 out of 16) was transferred to the ICU because of respiratory failure, which is reported in the literature as the main cause of ICU transfer [26,27,28,29]. Although a progress in therapy is being made and new practices in respiratory support, such as the use of high-flow nasal cannula, have been successfully implemented, respiratory failure remains the main cause of ICU admission in the course of RSV disease, and a variety of extrapulmonary complications, including neurological or cardiac sequelae, may also occur, leading to intubation; the risk rate for mechanical ventilation varies between 12.9 and 14.3 per 1000 RSV hospitalizations in children [30,31].

A large systematic review by Shi and colleagues investigated 20 potential risk factors for poor outcome of RSV ALRI, defining poor outcome as prolonged LOS, a need for oxygen supplementation, mechanical ventilation, and/or ICU admission [22]. The review identified 27 studies in children younger than 5 years and found a statistically significant association only with prematurity (OR = 1.75 for those born <37 weeks gestational age and OR = 2.68 for those born ≤32 weeks), younger age (OR = 4.91 for <3 months and OR = 2.02 for <6 months), comorbidities (OR = 2.69; 95% CI, 1.89–3.83), and congenital heart disease (OR = 3.40; 2.14–5.40) [22].

The risk factors for severe outcomes identified in our univariate analysis (but not in the multivariate model) included prematurity and younger age, which is consistent with the systematic review by Shi [22]. Similar to Shi, we observed an inverse relationship between gestational age and the odds of respiratory failure (OR = 0.81) and ICU transfer (OR = 0.82), as well as an inverse relationship between age and the odds of respiratory failure or ICU transfer, with a 43–46% decrease in odds per month of age. A recent study from Norway showed that respiratory support in patients with RSV infection was strongly associated with age < 3 months (aOR = 6.73), while in our group of patients, the odds were even higher (OR = 7.79 for respiratory failure, which in these patients meant the need for respiratory support) and OR = 8.37 for ICU transfer in those aged up to 3 months [23]. However, in contrast to the Norwegian study, we found no association with the number of siblings or comorbidities [23].

A high number of comorbidities were shown to be significant for ICU admission in the study by Cai, who showed that in children under 5 years of age, not only prematurity (OR = 6.71), younger age (<5 months, OR = 2.39), or low birth weight (OR = 6.77) but also an underlying respiratory and cardiovascular disease specific to the perinatal period, congenital defect originating in the perinatal period, congenital malformation of the heart and/or great vessels, cardiovascular disease, neurological disorder, and blood or liver disease [21]. These conditions should be treated with extreme caution, although even meta-analyses tend not to confirm their importance (as well as immunodeficiency) for the course of RSV disease [22]. For the general understanding of the disease, it is crucial to emphasize that due to the relatively low incidence of these chronic conditions in a population, the majority of severe cases occur in otherwise healthy infants; this was the case in our study. Although our research did not confirm the above-mentioned risk groups, it does not claim to undermine their importance or the knowledge on the subject; our results are probably the effect of the relatively low number of patients with these specific health conditions in our group. However, it is important to emphasize that a strong focus on comorbidities may lead to the misconception that otherwise healthy infants are not at risk for serious disease.

A large group of patients requiring special attention is related to prematurity; since this group is particularly vulnerable, prophylactic programs with the use of monoclonal antibodies have been widely introduced in national health systems [4]. In addition to its association with respiratory failure and ICU transfer, our univariate model shows that prematurity is also associated with higher odds of oxygen therapy. In our opinion, the need for oxygen supplementation reflects the severity of RSV disease even better than ICU transfer, since the majority of hospitalized infants do not develop respiratory failure, whereas many patients require standard oxygen therapy [31]. The need for oxygen therapy was also strongly related to clinical presentation and capillary blood gas parameters. Three groups of factors (the CBG, apnea, and pulse oximetry), in our opinion, deserve special attention with regard to the need for oxygen therapy, while others, including dyspnea, which is a typical presentation of a patient hospitalized due to RSV ALRI, or lack of fever, are less likely to play a role as single predictive factors; nevertheless, their inclusion in multifactorial assessment tools (scales or scoring systems, for example) could be successfully verified in future studies.

Apnea may be seen in the course of RSV disease, but the exact frequency of apnea is difficult to determine; a systematic review by Ralston reports an incidence ranging from 1.2% to 23.8%, with higher rates in preterm infants; in addition, apnea may be misdiagnosed in those with underlying neuromuscular disorders [32]. Our study shows an association not only between the presence of apnea and the likelihood of oxygen therapy but also with respiratory failure and ICU transfer. This is in agreement with the study by Kneyber who previously showed that apnea was associated with the higher number of ICU admissions due to mechanical ventilation and oxygen administration [33]. In addition, the risk of mechanical ventilation may increase with the number of apnea episodes [33]. Clinically, patients with apnea present with lower body temperature and worse blood gas parameters (i.e., lower pH and higher pCO_2_); apnea episodes were more frequent in younger patients, mostly those under 2 months of age [33]. In the group of 86 infants born before 34 weeks of gestation, very severe RSV disease (i.e., requiring invasive or non-invasive positive pressure ventilation) was associated with the presence of apnea [34], and in a general population of infants hospitalized in Shanghai, China, the proportion of apnea was higher in those with severe RSV ALRI [35]. The UK National Institute for Health and Care Excellence (NICE) guidelines on bronchiolitis recommend hospital admission for those with observed or reported apnea, while recurrent apnea episodes should raise suspicion of impending respiratory failure [36]. Our findings support the importance of apnea as a warning sign.

The NICE guidelines also recommend the use of pulse oximetry in any patient suspected of having bronchiolitis but emphasize that the importance of oxygen saturation should be reduced. This is because over-reliance on isolated pulse oximetry results may lead to over-referral to hospital or the use of unnecessary therapies or, conversely, to a reluctance to refer a patient to hospital on the basis of a normal oxygen saturation (i.e., above 92%) when the patient may be presenting with symptoms that would suggest hospital referral [36,37]. In this study, we found that pulse oximetry (%Sat) was reliably associated (in both univariate and multivariate models) with oxygen therapy but not with respiratory failure or ICU transfer, which seems to reflect its practical use; in fact, many studies and recommendations refer to pulse oximetry as one of the most widely accepted methods of oxygenation assessment in infants and children, mainly because of its safety, ease of use, low cost, wide availability, and repeatability when used by trained personnel [31,38,39].

In contrast, CBG parameters (pH, pCO_2_, and SatO_2_) were associated not only with a higher likelihood of oxygen therapy but also with the risk of respiratory failure and ICU admission. Capillary blood gas (CBG) appears to be a promising tool for assessing the risk of severe RSV disease progression, as it is an easily accessible, repeatable test that is now gaining popularity but is not widely used or recommended. The first studies on CBG date back to 50 years ago, and since then CBG has been shown to reliably reflect arterial blood acid-base balance parameters, whereas arterial blood monitoring is a more invasive method associated with more frequent complications (including hemorrhage, aneurysm, or thrombosis) and requires more complicated training of hospital staff [40,41,42,43]. Moreover, CBG has been shown to be independent of prior warming of the puncture site, patient hypo/hyperthermia, or capillary refill time over 3 s, and even under more difficult conditions, such as those found in ICU patients, the coefficients of correlation between capillary and arterial blood remain high (between r = 0.674 for pO_2_, r = 0.823 for pH, and r = 0.988 for pCO_2_) [40]. In our patient group, children who required supplemental oxygen therapy, developed respiratory failure, and/or required ICU transfer had worse CBG values (i.e., lower pH and SatO_2_ and higher pCO_2_) on admission. We have previously published the results of a CBG utility analysis in children hospitalized for bronchiolitis, showing that acidosis and hypercapnia adequately identify those at higher risk for ICU transfer [44]; similar results were obtained by Vo and colleagues, who showed that CBG pCO_2_ predicts respiratory decompensation in infants with bronchiolitis [45].

In this study, we also observed the relationship between CBG levels and prolonged LOS and pneumonia. Because prolonged LOS may be directly related to a worse clinical condition of patients, many studies have used it as a proxy for severe disease course; at the same time, it is also subject to many confounding factors, including local treatment practices, different accessibility to health care services, or local epidemiology, so it should be interpreted cautiously [22,23,46]. Except for CBG parameters, we found an association between LOS and low birth weight and a history of aspiration; history of aspiration might have led to pneumonia (as shown in the univariate and multivariate analyses on pneumonia). Low birth weight itself may reflect both a higher risk of poorer outcomes and physicians’ concern for the most vulnerable patients. Similarly, concern for the patient (as one of the factors) may partially explain the prolonged hospitalization of preterm infants observed in the univariate analysis. Nevertheless, in our series of patients, low birth weight was associated with prolonged LOS but not with the other endpoints, including respiratory failure or ICU transfer; this is in contrast to some previous studies that reported significant odds of poor outcome in children with low birth weight [22]. It should be noted, however, that we defined low birth weight as weight < 2500 g, whereas different studies used different definitions (also 1500 g or 10th), making direct comparisons difficult [22].

Pneumonia is one of the most important RSV-related concerns, as RSV can directly cause viral pneumonia or patients may be additionally co-infected with bacteria, and both can often lead to hospitalization and severe disease course, increasing the risk of ICU transfer, especially in at-risk groups [7,26,47,48,49]. For pneumonia, we found an association with older age, the aforementioned aspiration episode, clinical features including fever, duration of fever, duration of symptoms or presence of dyspnea, and increased inflammatory markers or hypercapnia; while this study does not pretend that any of these are diagnostic for pneumonia, further benefit could be gained from studies focusing on their possible use in RSV-infected children for the purpose of pneumonia prediction or risk stratification. We also found an association with maternal nicotine exposure, which shows possible effects of cigarette smoke exposure.

Special attention should be paid to two potentially modifiable factors that need to be discussed in detail: tobacco smoke exposure as a risk factor and a protective role of breastfeeding. Although tobacco smoke exposure has been identified as a risk factor and ample evidence has accumulated on the role of second-hand smoke exposure, studies on exposure during pregnancy are more complicated because of the possible delayed effect combined with a latency period that makes the effects less apparent; in addition, there are few methods of verification, and the most widely used one, i.e., anamnesis, may be misleading because, for example, mothers are unwilling to admit exposure to tobacco smoke or simply reduce exposure. Nevertheless, tobacco smoke has been shown to be a risk factor for RSV disease, hospitalization for RSV disease, and/or its severe course, and there are studies showing an increased risk of post-RSV wheezing due to prenatal tobacco smoke exposure [50,51,52,53,54,55,56]. We found an association between prenatal cigarette smoke exposure and pneumonia and with the need for oxygen therapy, in the latter case the effect was stronger when both parents smoked than when the father smoked alone, although, surprisingly, it was not significant in the case of a smoking mother. Because we relied on survey responses, these results are susceptible to bias, and other studies also show reluctance to answer questions about smoke exposure [56].

While tobacco smoke exposure was associated with an increased risk of oxygen therapy, breastfeeding in our group of patients was shown to have a protective effect in the multivariate regression model, which is consistent with previously published studies. A recent systematic review by Mineva and colleagues included 19 studies in infants younger than 12 months and concluded that non-breastfeeding practices were associated with a higher risk of severe RSV ALRI and hospitalization. The evidence included in the systematic review suggests a beneficial effect of breastfeeding in terms of reduced hospital admission, length of stay, supplemental oxygen requirements, and ICU admission [57]. We only observed the benefit in terms of lower odds of oxygen therapy, which supports the findings of a Korean retrospective analysis of infants hospitalized in four hospitals [58]. The Korean study showed the protective effect in terms of the rate of oxygen therapy (OR = 0.27), while the rate of ICU admission was lowest in the exclusively breastfed infants, but the differences were not statistically significant [58]. A Japanese study also showed a similar decrease in the odds ratio of oxygen therapy (OR = 0.26), while the decrease observed in our group was lower (OR = 0.49), and we did not detect effects on shorter LOS, as found in the Japanese study [59]. Similarly, the study by Dornelles showed an inverse relationship between the duration of breastfeeding and the duration of oxygen therapy and LOS [60]. In contrast to this study, we did not assess the duration of oxygen therapy, but only the need for oxygen therapy.

It must be recognized that there are other potential risk factors that did not reach statistical significance in our group. For example, socioeconomic factors and social vulnerability may also be risk factors, but in terms of increased RSV-related admissions rather than the course of the disease itself; the Fitzpatrick study showed that young maternal age, a criminal history, or a history of serious mental health/addiction problems may result in a higher risk of RSV hospitalization, but in our preselected group (the study was conducted in hospitalized children), we found no statistical significance for maternal age or crowding (expressed as the number of persons per household room) [61].

There are some strengths and limitations of this study. First, we conducted only a single-center study, and although the number of patients is relatively large, the generalizability of our results is subject to limitations. We believe that only huge meta-analyses can guarantee reliable conclusions. However, it is essential to provide this type of data because meta-analyses are based on the results from different sites and studies. Second, according to the principles of evidence-based medicine, the retrospective nature of this study ranks it lower than a prospective one; however, due to the structured questionnaires used in the medical records, the lack of data is not a significant problem here, and the retrospective design of the analysis did not influence patient management. Third, although our results do not confirm previously documented findings regarding certain health conditions, such as congenital heart disease or other comorbidities, we do not claim that these risk factors are not significant; the study simply reflects the daily clinical practice of a medium-size pediatric ward and reflects the general population, in which these conditions may not be seen, even with prolonged observation. An issue that was not addressed in this study was possible co-infection, especially with other viruses, while some studies suggested that co-infection (e.g., parainfluenza virus type 3) might increase the risk of a worse disease course [62]. Other intriguing questions are the type of respiratory support used and the long-term effects of RSV hospitalization in children with risk factors, both of which may also yield interesting results.

In conclusion, our study showed that younger age (up to 3 months), prematurity, and the presence of apnea are risk factors for a more severe course of the disease, as assessed by the need for oxygen therapy, the risk of respiratory failure, and ICU transfer. One of the suggestions that emerged from our study was a greater focus on apnea, which may play a crucial role in risk assessment and therefore require increased attention. The study also showed that two modifiable factors play a significant role: cigarette smoke exposure during pregnancy may influence disease severity, while breastfeeding is a protective factor.

## Figures and Tables

**Table 1:** Baseline characteristics of patients.

Characteristics	*n* (% of Group)	M ± SD/Me (Q1; Q3)
Gender, *n* (%)	594 (100)	
Female	244 (41.1)	
Male	350 (58.9)	
Age, months, mean ±SD		4.04 ± 4.56
Age group, *n* (%)	594 (100)	
Newborn	71 (12.0)	
2–3 months	315 (53.0)	
>3 months	208 (35.0)	
Week of pregnancy, weeks, median (Q1; Q3)		39.00 (38.00; 40.00)
Preterm, *n* (%)	83 (14.0)	
Birth weight, g, median (Q1; Q3)		3400.00 (3030.00; 3710.00)
Low birth weight (<2500 g), *n* (%)	54 (9.1)	
Apgar score, points, median (Q1; Q3)		10.00 (10.00; 10.00)
Apgar score, points, *n*(%)	570 (96.0)	
0–3 points	2 (0.3)	
4–7 points	27 (4.6)	
8–10 points	541 (91.1)	
No data	24 (4.0)	
Delivery during the RSV infection season, *n* (%)	422 (71.0)	
Nicotinism of mother, *n* (%)	50 (8.4)	
Exposure to nicotine during pregnancy, *n* (%)	553 (93.1)	
No exposure	426 (71.7)	
Mother	9 (1.5)	
Father	96 (16.2)	
Mother and Father	22 (3.7)	
No data	41 (6.9)	
Maternal age, years, mean ±SD		31.41 ± 4.69
Feeding method, *n* (%)	556 (93.6)	
Breastfeeding only	334 (56.2)	
Breastfeeding + modified milk	70 (11.8)	
Modified milk only	152 (25.6)	
No data	38 (6.4)	
Chronic diseases, *n* (%)		
Allergies	26 (4.4)	
Respiratory system—other disease	6 (1.0)	
Cardiological diseases	24 (4.0)	
Neurological diseases	5 (0.8)	
Down syndrome	1 (0.2)	
Immunological disorders	2 (0.3)	
Genetic diseases	1 (0.2)	
Social conditions, median (Q1; Q3):		
Number of persons in household		4.00 (3.00; 4.00)
Number of rooms in household		3.00 (2.00; 3.00)
Persons/room		1.50 (1.25; 2.00)
Sibling:		
No sibling, *n* (%)	175 (29.5)	
Number of siblings, median (Q1; Q3)		1.00 (0.00; 1.00)
Sibling 1, age, years, median (Q1; Q3)		3.00 (2.00; 4.00)
Sibling 2, age, years, median (Q1; Q3)		6.00 (4.00; 9.00)
Sibling 3, age, years, mean ±SD		11.00 ± 4.22
Sibling 4, age, years, mean ±SD		15.50 ± 6.95
Sibling 5, age, years *		8.00 *
No data, *n* (%)	25 (4.2)	
Fever duration, days, median (Q1; Q3):		
Before hospitalization,		0.00 (0.00; 1.00)
During hospitalization		0.00 (0.00; 0.00)
Total duration		0.00 (0.00; 2.00)
Fever, °C, *n* (%)	586 (98.7)	
No fever	355 (59.8)	
37.7 < 38	82 (13.8)	
38 < 39	109 (18.4)	
39 < 40	38 (6.4)	
>40.0	2 (0.3)	
No data	8 (1.3)	
Duration of symptoms, days, ±SD		4.03 ± 2.01
Symptoms, *n* (%):		
Cough	571 (96.1)	
Rhinitis	475 (80.0)	
Dyspnea	429 (72.2)	
Apnea	15 (2.5)	
Foamy saliva	230 (38.7)	
Aspiration	11 (1.9)	
Apathy	107 (18.0)	
Feeding difficulties	272 (45.8)	
Seizures	1 (0.2)	
Vomiting	93 (15.7)	
Diarrhea	51 (8.6)	
Infection at home, *n* (%)	272 (45.8)	
Respiratory rate per min, mean ±SD		55.68 ± 11.80
Saturation in pulse oximetry (%Sat), median (Q1; Q3)		96.00 (94.00; 97.00)
Heart Rate per min, mean ±SD		142.83 ± 17.42
Inflammatory markers, median (Q1; Q3):		
C-reactive protein (CRP), mg/L		1.61 (0.34; 5.56)
Procalcitonin (PCT), ng/mL		0.04 (0.00; 0.10)
White blood cells (WBC), 1 × 10^3^/µL		10.30 (8.30; 13.12)
Absolute neutrophil count (ANC), 1 × 10^3^/µL		2.20 (1.23; 4.17)
Capillary blood gas analysis (CBG):		
pH, median (Q1; Q3)		7.41 (7.39; 7.43)
Acidosis (pH < 7.35), *n* (%)	20 (3.4)	
pCO_2_, mmHg, median (Q1; Q3)		35.80 (32.70; 40.20)
Hypercapnia (pCO_2_ > 45 mmHg), *n* (%)	53 (8.9)	
Saturation (SatO_2_), %, median (Q1; Q3)		91.10 (88.20; 93.60)
Passive oxygen therapy, (pO2Tx), *n* (%)	197 (33.2)	
Passive oxygen therapy, day of hospitalization, median (Q1; Q3)		1.00 (1.00; 2.00)
Passive oxygen therapy, days, median (Q1; Q3)		0.00 (0.00; 2.00)
Pneumonia, *n* (%)	126 (21.2)	
Respiratory failure, *n* (%)	15 (2.5)	
Intensive care unit transfer, *n* (%)	16 (2.7)	
Hospitalization, days, median (Q1; Q3)		9.00 (8.00; 11.00)
Hospitalization time > median (9.00 days), *n* (%)	263 (44.3)	
Deaths, *n* (%)	0	

M—mean, SD—standard deviation, Me—median, Q1—1st quartile, Q3—3rd quartile, g—grams, °C—Celsius degree, pCO_2_—partial pressure of carbon dioxide. * Mean sibling 5 age, no standard deviation due to *n* = 1 observations.

**Table 2:** Univariate regression model analysis of risk factors for passive oxygen therapy.

	Univariate Regression Model		
Characteristics	OR	95%CI	*p*-Value
Gender, female vs. male	1.21	0.85–1.71	0.294
Age, month	0.98	0.94–1.02	0.391
Age ≤ 3 months (vs. >3 months)	**1.56**	**1.08–2.27**	**0.018**
Week of pregnancy, weeks	**0.91**	**0.85–0.98**	**0.013**
Preterm	**1.71**	**1.06–2.74**	**0.026**
Birth weight, g	**1.00**	**1.00–1.00**	**0.049**
Low birth weight (<2500 g)	1.71	0.96–3.01	0.063
Apgar score, points	1.01	0.85–1.23	0.873
Delivery during the RSV infection season	**1.72**	**1.17–2.59**	**0.007**
Exposure to nicotine during pregnancy (vs. no exposure):			
Mother	1.20	0.25–4.64	0.795
Father	**1.80**	**1.13–2.83**	**0.012**
Mother and Father	**2.41**	**1.01–5.76**	**0.046**
Maternal age, years	1.02	0.98–1.06	0.245
Feeding method:			
Breastfeeding vs. modified milk	0.68	0.46–1.02	0.064
Breastfeeding and modified milk vs. modified milk	1.18	0.66–2.10	0.577
Chronic diseases:			
Cardiological diseases	1.01	0.40–2.33	0.986
Neurological diseases	1.35	0.18–8.19	0.745
Down syndrome	N/A *	-	0.979
Social conditions (persons/room)	1.21	0.85–1.70	0.291
Number of siblings	0.99	0.79–1.22	0.898
Presence of fever	0.79	0.55–1.12	0.194
Fever duration, days	0.95	0.85–1.05	0.295
Duration of symptoms, days	0.92	0.84–1.01	0.081
Symptoms:			
Cough	1.82	0.72–5.59	0.242
Dyspnea	**5.09**	**3.14–8.66**	**<0.001**
Apnea	**5.81**	**1.96–21.19**	**0.003**
Aspiration	1.15	0.30–3.87	0.820
Saturation in pulse oximetry (%Sat), %	**0.78**	**0.73–0.84**	**<0.001**
Inflammatory markers:			
C-reactive protein (CRP), mg/L	1.00	0.99–1.01	0.579
Procalcitonin (PCT), ng/mL	0.83	0.51–1.11	0.318
White blood cells (WBC), 1 × 10^3^/µL	0.96	0.92–1.01	0.102
Absolute neutrophil count (ANC), 1 × 10^3^/µL	0.97	0.91–1.03	0.297
Capillary blood gas analysis (CBG):			
Acidosis (pH < 7.35)	1.08	0.40–2.68	0.872
pCO_2_, mmHg	**1.10**	**1.07–1.13**	**<0.001**
Hypercapnia (pCO_2_ > 45 mmHg)	**2.66**	**1.51–4.75**	**<0.001**
Saturation (SatO_2_), %	**0.87**	**0.83–0.91**	**<0.001**

OR—odds ratio, CI—confidence interval, g—grams, pCO_2_—partial pressure of carbon dioxide. * Not run due to insufficient number of events. Values in bold indicate statistically significant results.

**Table 3:** Multivariate regression model analysis of risk factors for passive oxygen therapy.

	Multivariate Regression Model		
Characteristics	OR	95%CI	*p*-Value
Gender, female vs. male	1.30	0.76–2.26	0.338
Age, month	1.02	0.89–1.16	0.748
Preterm	1.86	0.61–5.53	0.266
Low birth weight (<2500 g)	1.78	0.38–8.27	0.462
Apgar score, points	**1.60**	**1.06–2.58**	**0.035**
Delivery during the RSV infection season	1.25	0.61–2.61	0.549
Exposure to nicotine during pregnancy (vs. no exposure):			
Mother	1.49	0.04–64.55	0.827
Father	1.34	0.68–2.61	0.387
Mother and Father	2.32	0.17–51.10	0.547
Feeding method:			
Breast feeding vs. modified milk	**0.49**	**0.26–0.92**	**0.027**
Chronic diseases:			
Cardiological diseases	1.46	0.26–6.60	0.641
Neurological diseases	1.19	0.04–34.45	0.910
Down syndrome	N/A *	-	0.987
Presence of fever	1.01	0.43–2.38	0.976
Fever duration, days	0.96	0.72–1.26	0.762
Symptoms:			
Cough	0.45	0.10–2.44	0.314
Dyspnea	**5.22**	**2.43–12.51**	**<0.001**
Apnea	6.00	0.85–57.62	0.084
Aspiration	5.53	0.65–38.06	0.090
Saturation in pulse oximetry (%Sat), %	**0.83**	**0.75–0.92**	**<0.001**
Inflammatory markers:			
C-reactive protein (CRP), mg/L	1.01	0.99–1.04	0.458
Procalcitonin (PCT), ng/mL	0.90	0.38–1.52	0.767
White blood cells (WBC), 1 × 10^3^/µL	0.97	0.87–1.08	0.594
Absolute neutrophil count (ANC), 1 × 10^3^/µL	0.97	0.79–1.17	0.738
Capillary blood gas analysis (CBG):			
Acidosis (pH < 7.35)	0.31	0.05–1.62	0.183
Hypercapnia (pCO_2_ > 45 mmHg)	1.19	0.43–3.22	0.730
Saturation (Sat O_2_), %	**0.87**	**0.80–0.93**	**<0.001**

OR—odds ratio, CI—confidence interval, g—grams, pCO_2_—partial pressure of carbon dioxide. * Not run due to insufficient number of events. Values in bold indicate statistically significant results.

**Table 4:** Univariate regression model analysis results for risk factors for pneumonia.

	Univariate Regression Model		
Characteristics	OR	95%CI	*p*-Value
Gender, female vs. male	1.28	0.85–1.93	0.241
Age, month	**1.10**	**1.06–1.15**	**<0.001**
Age ≤ 3 months (vs. >3 months)	**0.45**	**0.30–0.67**	**<0.001**
Week of pregnancy, weeks	0.97	0.90–1.06	0.529
Preterm	1.37	0.78–2.31	0.260
Birth weight, g	1.00	1.00–1.00	0.602
Low birth weight (<2500 g)	1.10	0.54–2.09	0.789
Apgar score, points	1.04	0.85–1.31	0.754
Delivery during the RSV infection season	0.67	0.44–1.02	0.060
Exposure to nicotine during pregnancy (vs. no exposure):			
Mother	**5.01**	**1.30–20.64**	**0.018**
Father	1.34	0.78–2.22	0.273
Mother and Father	0.89	0.25–2.46	0.839
Maternal age, years	1.01	0.97–1.06	0.590
Feeding method:			
Breastfeeding vs. modified milk	0.98	0.61–1.61	0.943
Breastfeeding and modified milk vs. modified milk	0.93	0.44–1.88	0.838
Chronic diseases:			
Cardiological diseases	0.73	0.21–1.98	0.580
Neurological diseases	0.00	-	0.983
Down syndrome	N/A *	-	0.978
Social conditions (persons/room)	1.22	0.83–1.75	0.297
Number of siblings	1.27	1.00–1.61	0.051
Presence of fever	**3.92**	**2.59–5.99**	**<0.001**
Fever duration, days	**1.54**	**1.39–1.72**	**<0.001**
Duration of symptoms, days	**1.10**	**1.00–1.22**	**0.044**
Symptoms:			
Cough	1.29	0.47–4.51	0.648
Dyspnea	**1.62**	**1.02–2.66**	**0.045**
Apnea	0.56	0.09–2.08	0.456
Aspiration	**4.63**	**1.37–16.31**	**0.013**
Saturation in pulse oximetry (%Sat), %	0.95	0.89–1.02	0.178
Inflammatory markers:			
C-reactive protein (CRP), mg/L	**1.07**	**1.05–1.10**	**<0.001**
Procalcitonin (PCT), ng/mL	**1.44**	**1.09–2.07**	**0.027**
White blood cells (WBC), 1 × 10^3^/µL	**1.05**	**1.00–1.10**	**0.029**
Absolute neutrophil count (ANC), 1 × 10^3^/µL	**1.15**	**1.08–1.23**	**<0.001**
Capillary blood gas analysis (CBG):			
Acidosis (pH < 7.35)	2.05	0.76–5.13	0.134
pCO_2_, mmHg	0.98	0.95–1.01	0.277
Hypercapnia (pCO_2_ > 45 mmHg)	**2.06**	**1.10–3.73**	**0.020**
Saturation (SatO_2_), %	1.00	0.96–1.05	0.872

OR—odds ratio, CI—confidence interval, g—grams, pCO_2_—partial pressure of carbon dioxide. * Not run due to insufficient number of events. Values in bold indicate statistically significant results.

**Table 5:** Multivariate regression model analysis results for risk factors for pneumonia.

	Multivariate Regression Model		
Characteristics	OR	95%CI	*p*-Value
Gender, female vs. male	1.14	0.61–2.16	0.677
Age, month	0.99	0.87–1.12	0.873
Preterm	2.35	0.71–7.06	0.140
Low birth weight (<2500 g)	0.83	0.14–4.58	0.829
Apgar score, points	1.27	0.81–2.21	0.347
Delivery during the RSV infection season	1.06	0.49–2.38	0.876
Exposure to nicotine during pregnancy (vs. no exposure):			
Mother	2.83	0.03–629.00	0.686
Father	1.21	0.53–2.63	0.641
Mother and Father	4.51	0.15–437.27	0.445
Feeding method:			
Breastfeeding vs. modified milk	1.28	0.58–2.99	0.555
Chronic diseases:			
Cardiological diseases	0.26	0.03–1.49	0.172
Neurological diseases	0.00	-	0.993
Down syndrome	N/A *	-	0.995
Presence of fever	0.66	0.24–1.72	0.409
Fever duration, days	**1.70**	**1.31–2.26**	**<0.001**
Symptoms:			
Cough	1.44	0.23–16.93	0.731
Dyspnea	1.41	0.68–3.09	0.368
Apnea	0.00	-	0.983
Aspiration	**40.75**	**5.94–444.64**	**<0.001**
Saturation in pulse oximetry (%Sat), %	1.06	0.94–1.20	0.379
Inflammatory markers:			
C-reactive protein (CRP), mg/L	**1.06**	**1.02–1.10**	**0.001**
Procalcitonin (PCT), ng/mL	**0.61**	**0.38–0.95**	**0.030**
White blood cells (WBC), 1 × 10^3^/µL	0.97	0.85–1.10	0.631
Absolute neutrophil count (ANC), 1 × 10^3^/µL	1.16	0.94–1.44	0.173
Capillary blood gas analysis (CBG):			
Acidosis (pH < 7.35)	2.64	0.50–14.57	0.251
Hypercapnia (pCO_2_ > 45 mmHg)	**4.36**	**1.56–11.97**	**0.004**
Saturation (Sat O_2_), %	0.97	0.90–1.04	0.417

OR—odds ratio, CI—confidence interval, g—grams, pCO_2_—partial pressure of carbon dioxide. * Not run due to insufficient number of events. Values in bold indicate statistically significant results.

**Table 6:** Univariate regression model analysis of risk factors for respiratory failure.

	Univariate Regression Model		
Characteristics	OR	95%CI	*p*-Value
Gender, female vs. male	1.95	0.66–7.09	0.259
Age, month	**0.57**	**0.32–0.85**	**0.024**
Age ≤ 3 months (vs. >3 months)	**7.79**	**1.55–141.68**	**0.048**
Week of pregnancy, weeks	**0.81**	**0.72–0.93**	**0.001**
Preterm	**3.13**	**0.95–9.05**	**0.042**
Birth weight, g	1.00	1.00–1.00	0.061
Low birth weight (<2500 g)	2.57	0.57–8.42	0.153
Apgar score, points	0.74	0.56–1.07	0.054
Delivery during the RSV infection season	5.87	1.17–106.75	0.088
Exposure to nicotine during pregnancy (vs. no exposure):			
Mother	0.00	-	0.994
Father	1.11	0.25–3.59	0.870
Mother and Father	0.00	-	0.991
Maternal age, years	1.01	0.90–1.12	0.919
Feeding method:			
Breastfeeding vs. modified milk	1.38	0.40–6.26	0.636
Breastfeeding and modified milk vs. modified milk	2.22	0.40–12.29	0.335
Chronic diseases:			
Cardiological diseases	0.00	-	0.991
Neurological diseases	0.00	-	0.990
Down syndrome	0.00	-	0.993
Social conditions (persons/room)	1.09	0.37–2.38	0.845
Number of siblings	1.03	0.52–1.84	0.924
Presence of fever	**0.11**	**0.01–0.58**	**0.037**
Duration of symptoms, days	**0.60**	**0.40–0.85**	**0.008**
Symptoms:			
Cough	N/A *	-	0.991
Dyspnea	1.55	0.49–6.88	0.499
Apnea	**18.78**	**4.66–65.49**	**<0.001**
Aspiration	4.06	0.21–23.53	0.195
Saturation in pulse oximetry (%Sat), %	0.94	0.80–1.14	0.504
Inflammatory markers:			
Procalcitonin (PCT), ng/mL	0.74	0.02–1.50	0.741
White blood cells (WBC), 1 × 10^3^/µL	0.86	0.71–1.01	0.093
Absolute neutrophil count (ANC), 1 × 10^3^/µL	0.89	0.65–1.08	0.364
Capillary blood gas analysis (CBG):			
Acidosis (pH < 7.35)	4.74	0.71–18.92	0.051
pCO_2_, mmHg	**1.16**	**1.09–1.25**	**<0.001**
Hypercapnia (pCO_2_ > 45 mmHg)	**7.46**	**2.41–21.61**	**<0.001**
Saturation (SatO_2_), %	**0.88**	**0.82–0.94**	**<0.001**

OR—odds ratio, CI—confidence interval, g—grams, pCO_2_—partial pressure of carbon dioxide. * Not run due to insufficient number of events. Values in bold indicate statistically significant results.

**Table 7:** Multivariate regression model analysis of risk factors for respiratory failure.

	Multivariate Regression Model		
Characteristics	OR	95%CI	*p*-Value
Gender, female vs. male	4.41	0.84–38.29	0.114
Age, month	0.67	0.20–1.29	0.406
Preterm	3.03	0.12–30.91	0.401
Low birth weight (<2500 g)	0.46	0.00–37.20	0.725
Apgar score, points	0.66	0.32–1.75	0.278
Delivery during the RSV infection season	N/A *	-	0.994
Exposure to nicotine during pregnancy (vs. no exposure):			
Mother	0.67	-	>0.999
Father	0.59	0.11–3.21	0.582
Mother and Father	0.69	-	>0.999
Feeding method:			
Breastfeeding vs. modified milk	1.69	0.24–12.83	0.649
Chronic diseases:			
Cardiological diseases	0.00	-	0.998
Neurological diseases	0.00	-	0.999
Down syndrome	1.69	-	>0.999
Presence of fever	0.19	0.02–1.76	0.213
Symptoms:			
Cough	N/A *	-	0.998
Dyspnea	2.07	0.55–12.12	0.466
Apnea	**29.37**	**3.12–231.43**	**0.004**
Aspiration	0.00	-	0.998
Saturation in pulse oximetry (%Sat), %	1.03	0.79–1.30	0.849
Inflammatory markers:			
Procalcitonin (PCT), ng/mL	0.89	0.00–7.63	0.961
White blood cells (WBC), 1 × 10^3^/µL	0.79	0.53–1.05	0.202
Absolute neutrophil count (ANC), 1 × 10^3^/µL	1.51	0.87–2.68	0.134
Capillary blood gas analysis (CBG):			
Acidosis (pH < 7.35)	1.52	0.09–31.43	0.806
Hypercapnia (pCO_2_ > 45 mmHg)	0.71	0.10–5.20	0.752
Saturation (SatO_2_), %	0.86	0.72–1.01	0.075

OR—odds ratio, CI—confidence interval, g—grams, pCO_2_—partial pressure of carbon dioxide. * Not run due to insufficient number of events. Values in bold indicate statistically significant results.

**Table 8:** Univariate regression model analysis of risk factors for ICU transfer.

	Univariate Regression Model		
Characteristics	OR	95%CI	*p*-Value
Gender, female vs. male	2.13	0.73–7.69	0.195
Age, month	**0.54**	**0.30–0.82**	**0.017**
Age ≤ 3 months (vs. >3 months)	**8.37**	**1.68–151.88**	**0.040**
Week of pregnancy, weeks	**0.82**	**0.72–0.94**	**0.002**
Preterm	2.84	0.87–8.03	0.059
Birth weight, g	1.00	1.00–1.00	0.087
Low birth weight (<2500 g)	2.37	0.53–7.65	0.189
Apgar score, points	0.75	0.57–1.09	0.070
Delivery during the RSV infection season	6.30	1.26–114.41	0.076
Exposure to nicotine during pregnancy (vs. no exposure):			
Mother	0.00	-	0.994
Father	1.02	0.23–3.26	0.970
Mother and Father	0.00	-	0.991
Maternal age, years	1.00	0.90–1.11	0.975
Feeding method:			
Breastfeeding vs. modified milk	1.53	0.46–6.91	0.521
Breastfeeding and modified milk vs. modified milk	2.22	0.40–12.29	0.335
Chronic diseases:			
Cardiological diseases	0.00	-	0.991
Neurological diseases	0.00	-	0.990
Down syndrome	0.00	-	0.993
Social conditions (persons/room)	1.02	0.35–2.21	0.959
Number of siblings	1.04	0.54–1.82	0.905
Presence of fever	**0.11**	**0.01–0.53**	**0.031**
Duration of symptoms, days	**0.69**	**0.48–0.94**	**0.028**
Symptoms:			
Cough	N/A *	-	0.991
Dyspnea	1.16	0.40–4.19	0.802
Apnea	**17.18**	**4.30–59.06**	**<0.001**
Aspiration	3.79	0.20–21.77	0.218
Saturation in pulse oximetry (%Sat), %	0.94	0.80–1.14	0.506
Inflammatory markers:			
C-reactive protein (CRP), mg/L	1.01	0.97–1.02	0.623
Procalcitonin (PCT), ng/mL	0.72	0.02–1.48	0.721
Absolute neutrophil count (ANC), 1 × 10^3^/µL	0.91	0.68–1.09	0.433
Capillary blood gas analysis (CBG):			
Acidosis (pH < 7.35)	4.40	0.66–17.37	0.062
pCO_2_, mmHg	**1.16**	**1.09–1.25**	**<0.001**
Hypercapnia (pCO_2_ > 45 mmHg)	**6.70**	**2.20–18.88**	**<0.001**
Saturation (SatO_2_), %	**0.87**	**0.81–0.94**	**<0.001**

OR—odds ratio, CI—confidence interval, g—grams, pCO_2_—partial pressure of carbon dioxide. * Not run due to insufficient number of events. Values in bold indicate statistically significant results.

**Table 9:** Multivariate regression model analysis of risk factors for ICU transfer.

	Multivariate Regression Model		
Characteristics	OR	95%CI	*p*-Value
Gender, female vs. male	8.31	1.28–93.07	0.048
Age, month	0.29	0.04–1.07	0.144
Preterm	5.18	0.19–80.78	0.248
Low birth weight (<2500 g)	0.74	0.00–118.75	0.904
Apgar score, points	0.54	0.21–1.80	0.226
Delivery during the RSV infection season	N/A *	-	0.995
Exposure to nicotine during pregnancy (vs. no exposure):			
Mother	0.04	-	>0.999
Father	0.52	0.05–3.49	0.532
Mother and Father	0.56	-	>0.999
Feeding method:			
Breastfeeding vs. modified milk	7.37	0.40–706.55	0.283
Chronic diseases:			
Cardiological diseases	0.00	-	0.998
Neurological diseases	39,331.29	-	>0.999
Down syndrome	13,371.51	-	>0.999
Presence of fever	0.02	0.00–0.58	0.067
Symptoms:			
Cough	N/A *	-	0.999
Dyspnea	0.86	0.12–8.35	0.887
Apnea	**21.99**	**0.98–562.13**	**0.046**
Aspiration	0.00	-	0.999
Saturation in pulse oximetry (%Sat), %	1.02	0.72–1.47	0.913
Inflammatory markers:			
C-reactive protein (CRP), mg/L	**1.17**	**1.06–1.33**	**0.007**
Procalcitonin (PCT), ng/mL	0.02	0.00–0.54	0.311
Absolute neutrophil count (ANC), 1 × 10^3^/µL	1.16	0.68–1.81	0.537
Capillary blood gas analysis (CBG):			
Acidosis (pH < 7.35)	3.19	0.03–235.58	0.574
Hypercapnia (pCO_2_ > 45 mmHg)	0.22	0.01–2.31	0.281
Saturation (SatO_2_), %	**0.83**	**0.68–0.98**	**0.034**

OR—odds ratio, CI—confidence interval, g—grams, pCO_2_—partial pressure of carbon dioxide. * Not run due to insufficient number of events. Values in bold indicate statistically significant results.

**Table 10:** Univariate regression model analysis of risk factors for length of stay.

	Univariate Regression Model		
Characteristics	OR	95%CI	*p*-Value
Gender, female vs. male	0.83	0.59–1.16	0.269
Age, month	0.97	0.94–1.01	0.173
Age ≤ 3 months (vs. >3 months)	1.15	0.82–1.62	0.426
Week of pregnancy, weeks	**0.91**	**0.84–0.98**	**0.010**
Preterm	**1.76**	**1.09–2.86**	**0.021**
Birth weight, g	**1.00**	**1.00–1.00**	**0.028**
Low birth weight (<2500 g)	**2.89**	**1.59–5.49**	**<0.001**
Apgar score, points	0.84	0.69–1.00	0.061
Delivery during the RSV infection season	1.28	0.89–1.84	0.186
Exposure to nicotine during pregnancy (vs. no exposure):			
Mother	1.00	0.24–3.81	0.995
Father	1.12	0.71–1.75	0.619
Mother and Father	1.80	0.76–4.44	0.187
Maternal age, years	1.02	0.99–1.06	0.224
Feeding method:			
Breastfeeding vs. modified milk	0.80	0.54–1.18	0.259
Breastfeeding and modified milk vs. modified milk	1.26	0.71–2.25	0.432
Chronic diseases:			
Cardiological diseases	1.02	0.44–2.31	0.967
Neurological diseases	0.80	0.10–4.86	0.807
Down syndrome	N/A *	-	0.980
Social conditions (persons/room)	1.20	0.87–1.68	0.263
Number of siblings	1.15	0.94–1.42	0.177
Presence of fever	**1.51**	**1.08–2.12**	**0.016**
Fever duration, days	1.08	0.98–1.18	0.124
Duration of symptoms, days	**0.85**	**0.77–0.93**	**<0.001**
Symptoms:			
Cough	0.52	0.21–1.21	0.135
Dyspnea	1.41	0.97–2.04	0.071
Apnea	2.45	0.76–9.25	0.148
Aspiration	**4.93**	**1.22–32.82**	**0.045**
Saturation in pulse oximetry (%Sat), %	0.98	0.93–1.05	0.612
Inflammatory markers:			
C-reactive protein (CRP), mg/L	1.01	1.00–1.02	0.235
Procalcitonin (PCT), ng/mL	1.28	0.97–1.89	0.140
White blood cells (WBC), 1 × 10^3^/µL	1.01	0.97–1.05	0.523
Absolute neutrophil count (ANC), 1 × 10^3^/µL	1.05	0.99–1.11	0.095
Capillary blood gas analysis (CBG):			
Acidosis (pH < 7.35)	2.44	0.93–7.09	0.079
pCO_2_, mmHg	**1.04**	**1.01–1.06**	**0.011**
Hypercapnia (pCO_2_ > 45 mmHg)	**2.24**	**1.22–4.24**	**0.011**
Saturation (SatO_2_), %	**0.96**	**0.92–0.99**	**0.019**

OR—odds ratio, CI—confidence interval, g—grams, pCO_2_—partial pressure of carbon dioxide. * Not run due to insufficient number of events. Values in bold indicate statistically significant results.

**Table 11:** Multivariate regression model analysis of risk factors for length of stay.

	Multivariate Regression Model		
Characteristics	OR	95%CI	*p*-Value
Gender, female vs. male	0.84	0.54–1.31	0.443
Age, month	0.94	0.84–1.04	0.227
Preterm	0.79	0.29–2.05	0.627
Low birth weight (<2500 g)	2.70	0.72–10.31	0.141
Apgar score, points	0.93	0.67–1.27	0.643
Delivery during the RSV infection season	1.38	0.77–2.51	0.285
Exposure to nicotine during pregnancy (vs. no exposure):			
Mother	0.59	0.03–10.19	0.708
Father	1.20	0.67–2.13	0.530
Mother and Father	1.64	0.17–18.83	0.672
Feeding method:			
Breastfeeding vs. modified milk	0.85	0.50–1.46	0.561
Chronic diseases:			
Cardiological diseases	0.93	0.27–2.98	0.909
Neurological diseases	1.55	0.06–43.26	0.768
Down syndrome	1.07 × 10^5^	-	0.983
Presence of fever	1.42	0.71–2.83	0.322
Fever duration, days	1.10	0.88–1.36	0.405
Symptoms:			
Cough	0.46	0.12–1.68	0.236
Dyspnea	1.69	0.99–2.94	0.056
Apnea	0.85	0.13–5.46	0.862
Aspiration	4.97	1.01–36.78	0.067
Saturation in pulse oximetry (%Sat), %	0.98	0.90–1.07	0.606
Inflammatory markers:			
C-reactive protein (CRP), mg/L	1.01	0.99–1.03	0.303
Procalcitonin (PCT), ng/mL	0.89	0.61–1.41	0.563
White blood cells (WBC), 1 × 10^3^/µL	0.92	0.83–1.00	0.061
Absolute neutrophil count (ANC), 1 × 10^3^/µL	1.17	1.00–1.38	0.059
Capillary blood gas analysis (CBG):			
Acidosis (pH < 7.35)	1.10	0.25–5.24	0.900
Hypercapnia (pCO_2_ > 45 mmHg)	1.96	0.84–4.68	0.124
Saturation (SatO_2_), %	0.97	0.92–1.03	0.343

OR—odds ratio, CI—confidence interval, g—grams, pCO_2_—partial pressure of carbon dioxide.

## Data Availability

Data sets generated in this study are available from the corresponding author on request.
